# Supplementary material for: Are carbon emissions trading and green financial instruments synergistic? -Comprehensive quantitative research based on content analysis
Source: PLoS One. 2024 Mar 7;19(3):e0298601. doi: 10.1371/journal.pone.0298601 (PMC10919601; doi:10.1371/journal.pone.0298601)
Supplement: S1 Data — (ZIP) [file pone.0298601.s001.zip › Data/地级市Z_F工作报告.zip!/╔╜╢1⁄2/╢1⁄2╙¬╩╨/└┤╫╘╣1⁄2╓┌║┼í╛┬φ┐╦╩2╛▌═°í┐_2006.docx]

各位代表：

现在，我代表市人民政府向大会作政府工作报告，请予审议，并请市政协委员和其他列席人员提出意见。

一、“十五”时期政府工作回顾

过去的五年是很不平凡的五年。全市上下在省委、省政府和市委的正确领导下，在历届市领导班子打下的良好基础上，高举邓小平理论和“三个代表”重要思想伟大旗帜，认真贯彻党的十六大和十六届三中、四中、五中全会及市三次党代会精神，以科学发展观统领全局，解放思想，干事创业，改革开放和现代化建设取得了巨大成就。

五年来，全市经济快速发展，综合实力显著增强。初步统计，2005年全市生产总值突破千亿元，达到1166亿元，年均增长14.4%，高于“十五”计划预期7.4个百分点。农业和农村经济全面发展，产业化、标准化水平不断提高，60%多的农户进入产业链经营；畜牧、水产、蔬菜、冬枣、棉花等优势产业快速发展，一批区域化布局、规模化生产的农产品产业区正在形成；五年开发荒碱地46万亩，开发整理土地50万亩，实施农业综合开发66万亩，农业综合生产能力明显提高。实施工业强市战略取得显著成效，石油工业稳步发展，山东加工制造业基地建设快速推进，八大支柱产业初具规模。去年全市规模以上工业实现销售收入1585.8亿元、增加值865.7亿元，分别是“九五”末的2.9倍、2.7倍；实现利润458亿元、利税585.8亿元，分别是“九五”末的2.5倍、2.4倍；高新技术产业产值占全市规模以上工业总产值的比重由“九五”末的3.1%提高到13.5%；全市规模以上工业企业由187家增加到452家，其中销售收入过亿元的由27家增加到104家，过10亿元的由1家增加到20家；原油加工能力由400万吨增加到1500万吨，造纸能力由50万吨增加到120万吨，轮胎生产能力由650万条增加到1300万条，纺织能力由40万锭增加到120万锭，成为有影响力的石化基地、造纸强市、轮胎大市。服务业发展步伐加快，在石油价格急剧攀升和工业高速增长的情况下，增加值占全市生产总值的比重比“九五”末提高1.7个百分点。投资、消费不断扩大，五年完成全社会固定资产投资1847亿元，是“九五”的2.6倍，油田和地方投资比重由67.3：32.7变为19：81；去年社会消费品零售总额达到149亿元，年均增长16.9%。非公有制经济蓬勃发展，占全市生产总值的比重达到30%。地方财政收入较快增长，去年达到38.5亿元，年均增长21.1%。金融体系不断壮大，去年末全市金融机构各项存款余额652亿元、贷款余额420亿元，年均分别增长18.9%、24.1%，金融生态环境居全国第12位。

五年来，各项改革不断深化，对外开放实现重大突破。实施“大开放、大招商、大发展”战略取得丰硕成果，五年引进外来固定资产投资334亿元；实际利用外资6.2亿美元，是“九五”的4.9倍。对外经贸快速增长，去年完成进出口总值14.8亿美元，其中出口8.5亿美元，分别是“九五”末的10.3倍、11.4倍，在全省的排名五年分别前进了3个和5个位次。连续三年成功举办了东营经贸洽谈会。工业园区建成区面积达到87平方公里。大开放、大招商有力地促进了经济社会发展，促进了干部群众的思想解放和素质提高，促进了发展环境的改善，促进了东营知名度的提升。企业改革不断深化，乡及乡以上国有、集体企业改制基本完成，上市企业达到3家。国有资产监管体系基本建立。财政改革力度加大，推行了部门预算，建立完善了政府采购、财政集中支付等制度。投资、金融、价格等改革全面深化。粮食、棉花流通体制改革扎实推进，建立了地方粮食储备体系，全面放开了粮食市场。政府机构改革进展顺利，事业单位改革逐步展开。整顿和规范市场经济秩序工作扎实开展，食品、药品、农资、土地市场秩序和开发区、固定资产投资项目等专项治理整顿取得明显成效。

五年来，城市和基础设施建设力度不断加大，对经济社会发展的带动作用和承载能力进一步增强。城市规划设计水平不断提高，规划体系日趋完善。城市建设改造步伐加快，五年投入36.3亿元将中心城原有193.4公里道路全部改造一遍，并新建道路256.8公里，形成了十纵八横的城市发展框架；建成了一大批市政公用设施，城市功能进一步完善；东西城对接和城中村改造加快推进，中心城建成区面积由50平方公里扩大到100平方公里以上；五年新增绿地891万平方米，新建公园5个，治理城区河道191公里，城市水系进一步完善，绿化覆盖率由23%提高到36%，获山东省适宜人居环境奖，被命名为山东省园林城市。城市管理体制进一步理顺，综合执法顺利实施，环境综合整治扎实有效，城市面貌明显改观。城市建设投融资渠道不断拓宽，城区土地“五统一”管理深入实施，五年全市实现土地资产收益37.1亿元。县城和小城镇快速发展，全市城镇化水平由47.7%提高到58%。基础设施建设进一步加强。投资10.9亿元建成了利津、东营两座黄河公路大桥；投资16.6亿元建设改造干线公路520多公里；东营港扩建工程启动实施；改造完善了东营永安机场并已投入使用，初步形成了海陆空立体交通大格局。投资6亿多元建成了81.4公里的城东和垦东防潮大堤，构筑起了一道沿海安全屏障。城乡电网改造全面完成，供电保障能力大大增强。“数字化东营”建设走在全国前列，建成了城域网，开发建设了一批应用系统和平台，经济社会发展的信息化水平不断提高。

五年来，统筹城乡发展取得明显成效，农村生产生活条件得到很大改善。五年市财政支持基层和“三农”的资金达19亿元，相当于建市以来前17年总和的3.8倍。扶持农业龙头企业发展，五年市财政拿出9000万元扶持项目97个。加强农村基础设施建设，投资6.3亿元，在全国率先完成了大中型灌区节水改造，全市395公里干渠全部实现了高标准衬砌，所有农田基本达到了水浇条件；投资4.5亿元，治理骨干河道520多公里，提高了防洪排涝排碱能力；投资2.9亿元，新建水源工程306个，铺设供水管线2850公里，实现了村村通自来水，使全市百万农民告别了吃苦咸水、涮街水的历史，在此基础上又投资9081万元，实施城乡供水一体化工程，已使42万多农民用上了符合国家卫生标准的自来水；投资11.5亿元，建设改造农村公路3635公里，实现了村村通柏油路、通客车，解决了农民出行难的问题，改变了“晴天一身土、雨天一身泥”的状况；投资1.2亿元建设改造5处渔港，促进了渔业发展，为渔民提供了安全保障；大力推行“一池三改”，投资2200万元，使2.4万农户用上了安全洁净的沼气。改善农村消费环境，投资6000多万元，对乡镇首集市场全部进行了改造；投资7016万元，实施了超市进乡镇、放心店进村、检验检测进市场、“12315”进农村和建设药品配送网、食品药品安全监管网“四进两网”工程。加快农村教育发展，五年市财政投入农村教育的资金达4.3亿元，是建市以来前17年总和的8.1倍，全市农村中小学已全部消灭了危房，全部达到省级规范化二类建设标准，并实现了微机联网校校通。改善农村卫生条件，投资2496万元对乡镇卫生院全部改造了一遍，并为每个乡镇卫生院配备了高标准救护车，建设了规范化产科和预防接种门诊；投资729万元，建设了200个农村社区卫生服务站；市财政拿出780万元，对乡镇卫生院进行了经费补贴；实行药品集中招标采购和统一配送，降低了药品价格，让广大群众用上了放心药，基本解决了看病难的问题，看病贵的问题也得到了缓解。扎实开展小康文明村建设，已建成小康文明村178个。建设了一批乡镇文化中心和村文化大院，实现了村村通有线电视，丰富了群众的精神文化生活。加强村庄改造，投资450万元编制完成了900个村庄规划，实施了农村绿化工程，改善了村容村貌。深入开展千村万户农民培训，近两年投资712万元培训农民38.7万人。大力减轻农民负担，全部免征农业税，免除农村学校义务教育阶段在校生学杂费、课本费和作业本费，免收城乡集贸市场管理费；取消了“两工”，停征了乡村公益事业金，使全市百万农民彻底摆脱了延续几千年的“皇粮国税”，并对种粮农民、良种、农机具购置实行了补贴。对取消农业税减少的收入，全部由各级财政转移支付，保证了乡村组织正常运转。我市统筹城乡发展的做法得到了上级的充分肯定，省里连续两年在我市召开“三农”工作现场会，中央领导批示要求认真总结我市经验，在全国推广。

五年来，构建起以“五保五救助”为主要内容的保障救助体系，为贫困群众和弱势群体撑起了“保护伞”。五年市财政用于社会保障救助的资金达3.98亿元。城镇职工养老保险费征缴率达到98.5%，农村养老保险适龄参保率达到52%。城乡低保实现了应保尽保，保障水平不断提高，并对城市低保和特困家庭在用水、供热等方面实行了救助。建起了覆盖全市城镇职工、农民、城镇居民的三张医疗保险网，并每年分别拿出420万元和400万元建立了农民和城镇居民大病救助制度，较好地解决了群众看不起病和因病致贫、因病返贫的问题。城镇职工失业保险费征缴率达到96%。投资3502万元，改造和扩建乡镇敬老院39处，农村五保对象实现了集中供养，供养标准去年达到每人每年2500元，并全部由各级财政承担，同时市财政每年给每位五保对象发放400元的“零用钱”。实施教育救助，对城市低保户子女免除基础教育阶段学杂费等费用，城乡低保户子女考入大学的本科市里救助1万元、专科救助5000元，其他考入大学的贫困学生酌情救助；对在国办基础教育学校就读的盲、聋残疾学生免费提供上学机会，对弱智儿童实施免费义务教育；对低保户子女等贫困寄宿生进行生活补贴。实施残疾人救助，市财政每年拿出500万元，为重度特困残疾人每人每月发放80元生活补助，并帮助残疾人解决生产生活、康复医疗和上学等方面的困难。加强灾害救助，五年市里下拨救灾资金3660万元，确保了受灾群众有饭吃、有衣穿、有房住。实施老年人救助，为75岁以上农村老年人每人每年发放生活救助金360元。实施住房救助，建立了城镇最低收入家庭廉租住房制度，实施了城乡居民安居工程，五年投入3421万元为农村无房户、危房户建设住房1555套。“五保五救助”体系的建立，使广大群众特别是困难群众和弱势群体不再为基本生活而担忧，看病象干部职工一样能按比例报销，学生不再因贫困而辍学，老人们安度晚年，基本实现了老有所养、病有所医、失有所助、贫有所帮、灾有所救、居者有其屋。广大群众实实在在地感受到了社会主义大家庭的温暖，和谐之花在东营大地处处绽放!

五年来，油地军校结合日益深入，区域经济社会一体化快速推进。油地军校团结发展的思想深入人心，区域资源整合步伐加快，凝聚起了共建美好家园的强大合力。五年来，油地校共同招商引资，联手发展民营经济，联合推动区域科技进步，取得了很大成效。油地共同投资，实施了防潮堤建设、中心城道路建设改造、东城管道气工程、垃圾无害化处理场等一大批城市和基础设施项目，有力地改善了区域发展和群众生活环境。油田帮扶乡镇工作成效显著，五年投资2亿多元，实施帮扶项目171个。支持油军校加快改革与发展，改善职工生活条件。制定政策措施，规划建设油田工业园，支持油田存续企业改制和发展壮大，目前油田存续企业已完成改制25家，有30个项目入园建设。加快县域经济发展，广饶县跨入了全国百强县(市)行列，其他各县区都实现了快速发展。现在，油地军校亲如一家,县域经济竞相发展,东营大地呈现出欣欣向荣、蒸蒸日上的可喜局面!

五年来，各项社会事业全面进步，社会文明程度有了很大提高。深入实施科教兴市、人才强市战略，科技对经济增长的贡献率不断提高。全面落实教育优先发展的方针，义务教育水平进一步提高，基本普及了学前三年教育和高中阶段教育，创办了东营职业学院和东营市技术学院。油田中小学移交工作顺利完成。加强疾病预防控制和医疗救治体系建设，公共卫生服务水平和突发性公共卫生事件应急处理能力显著增强。广泛开展全民健身运动，竞技体育实现新突破，在全国十运会上我市运动员取得了2金1银的好成绩。人口和计划生育工作整体水平不断提高，年均人口自然增长率为5.87‰。能源资源节约和发展循环经济取得初步成效，生态市建设规划启动实施，污染防治力度加大，单位生产总值主要污染物排放量大幅度下降，城市空气质量日报良好率达100%；全市林木覆盖率由“九五”末的12%提高到20.6%，大片荒碱地正在变成良田和绿地。国防动员、人民防空、民兵预备役和双拥工作进一步加强。外事侨务、民族宗教、对台、档案、统计、史志、无线电管理等工作取得新成绩。妇女儿童、老年人、残疾人权益得到保障。地震、气象预测预报手段和水平进一步提高。思想道德建设不断深入，群众性精神文明创建活动广泛开展，创建文明城市和“信用东营”建设扎实有效。基层民主不断扩大，普法教育和依法治市深入实施。

五年来，“平安东营”建设扎实推进，确保了社会政治持续稳定。始终把维护稳定放在压倒一切的位置来抓，连续三年将“平安东营”建设列入“双十”工程，投资6484万元，完善了治安管理体系，改善了治安管理和案件侦破手段。建立健全矛盾纠纷排查调处机制，依法加强信访工作，及时化解各类矛盾，加强社会和油区治安综合治理，严厉打击各种犯罪行为，保持了良好的社会和油区秩序。毫不放松地抓好安全生产，安全生产事故主要指标连续五年全面下降，成为全省唯一连续三年无重特大事故的市。五年来，全市一直保持了“三个没有”：即没有发生影响重大的到省进京滋事事件，没有发生影响重大的群体性事件，没有发生影响重大的治安灾害事故，开创了社会政治持续稳定的良好局面。

五年来，城乡居民收入较快增长，生活质量和水平大幅度提高。五年新增就业13.8万人，城镇登记失业率始终控制在2%以内。全市城镇居民人均可支配收入按可比口径由“九五”末的7622元增加到14939.3元，年均增长14.4%；农民人均纯收入由2784元增加到4602.7元，年均增长10.6%；城乡居民人均储蓄由10009元增加到21398元。城市居民人均住房使用面积由“九五”末的13.3平方米提高到20.8平方米，农村居民人均居住面积由25.2平方米提高到27.9平方米。城镇每百户居民拥有电话290部、电脑59台，农村每百户居民拥有电话151.6部、电视机92.8台、摩托车71.2辆。全市汽车保有量达到11.7万辆，比“九五”末增加7万辆；家庭轿车达到3.4万辆。五年来，群众的餐桌越来越丰盛，衣着越来越漂亮，彩电已基本普及，手机、电脑不再是奢侈品，越来越多的人住上了新房、圆了汽车梦，全市人民的生活正在向更加宽裕的小康迈进!

五年来，政府职能加快转变，行政能力和水平有了新的提高。根据市场经济发展的要求，在改进经济调节、市场监管的同时，加强了社会管理和公共服务职能。认真贯彻行政许可法和国务院全面推进依法行政实施纲要，扎实推进法治政府、阳光政府、责任政府和服务政府建设，依法行政水平进一步提高。大力推进政府工作提速，深化行政审批制度改革，市级行政审批事项由“九五”末的600项精简到164项，普遍实行了行政许可集中、联合、统一办理制度，建立健全了政务公开、服务承诺等制度，政府工作效率和服务水平不断提高。加强公务员队伍建设，提高了整体素质，增强了责任感、使命感。坚持从严治政，加强政风建设，深入开展反腐败斗争，认真纠正部门和行业不正之风，严肃查处违纪违法案件，廉政建设得到进一步加强。

　　刚刚过去的2005年，全市人民团结一致，抢抓机遇，埋头苦干，奋发进取，胜利完成了市五届人大三次会议确定的各项主要任务，实现了经济社会又快又好发展。初步统计，全市生产总值比上年增长17.7%，全社会固定资产投资增长34.4%，社会消费品零售总额增长15.7%，外贸出口增长41.4%，地方财政收入增长36.1%，城镇居民人均可支配收入增长15.5%，农民人均纯收入增长14.1%，城镇登记失业率为1.9%，人口自然增长率为5.3‰，主要指标增幅高于“十五”时期平均增长速度，为实施“十一五”规划奠定了坚实基础。

各位代表，过去的五年是全市经济发展最快的五年，是改革开放成效最显著的五年，是统筹发展最好的五年，是城乡面貌变化最大的五年，是人民群众得实惠最多的五年。这五年，全市的经济总量、工业总产值、工业利税、固定资产投资、社会消费品零售总额、进出口总值、财政总收入及地方财政收入、城镇居民人均可支配收入、城乡居民储蓄余额、中心城建成区面积等十几项主要指标翻了一番多，五年的发展成果超过了建市以来前17年的总和，五年再造了一个新东营!这五年，我市综合实力大幅度提升，在全国百强城市中由2002年的49位跃升到2004年的27位，并跨入中国投资(硬)环境50优城市行列!这五年，我市成为国家卫生城市、国家环保模范城市、全国双拥模范城市、国家技术创新工程示范城市、国家水土保持生态环境建设示范城市、全国科技进步先进城市、国家级生态示范区、创建全国文明城市工作先进城市和省级文明城市!这五年，我市率先基本实现了“三免五通五保五救助”，许多工作走在了全国、全省的前列!今天的东营，经济快速发展，社会和谐稳定，人民群众安居乐业，到处充满了生机和活力，我们已经站在了一个新的历史起点上!

过去的五年，面对“入世”带来的挑战，国内外市场变化带来的影响，社会转型时期矛盾增多带来的压力，非典、禽流感等重大疫情和自然灾害带来的威胁，全市上下同心同德，众志成城，艰苦奋斗，迎难而上，经受住了一次又一次考验，取得了一个又一个胜利，开创了各项工作的新局面，充分展现了东营人民勤劳智慧、创新敬业、能拼善赢的良好风貌!

过去的五年，我们在实践中探索，在创新中前进，积累了丰富的经验：一是坚持以科学发展观统领全局，紧紧扭住发展不放松，加快发展、科学发展、和谐发展，依靠发展解决遇到的矛盾和问题。二是坚持把上级指示精神与东营实际结合起来，创造性地开展工作，丰富完善了“五位一体”的发展路子；准确把握经济社会发展趋势，注重研究解决深层次矛盾和问题，增强了工作的预见性。三是坚持把投入作为拉动发展的着力点，千方百计拓宽融资渠道，持续不断地加大方方面面的投入，有力地推动了经济总量膨胀、经济结构优化和各项社会事业的发展。四是坚持立足当前、着眼长远，既解决好经济社会发展和群众生产生活中的现实问题，又着力培育主导产业，加强重大基础设施建设，为长远发展奠定了坚实的基础。五是坚持执政为民，始终把为群众谋利益作为一切工作的出发点和落脚点，扎扎实实为群众办实事、办好事，每年实施“双十”工程，五年共投入117.8亿元，实施项目210个，有力地改善了群众生产生活条件；正确把握改革发展的方向，让广大群众在改革中得到实惠。六是坚持求真务实抓落实，建立完善决策目标、执行责任、考核监督“三个体系”，严格执行决策规则和程序，实行目标责任制，加强督查考核，确保了决策科学，执行有力，各项工作落到实处。七是坚持正确处理改革发展稳定的关系，全力维护社会稳定，加强全市上下、油地军校的团结，建立干事创业的激励机制，在全市形成了风正气顺心齐、团结和谐稳定的浓厚氛围。这些经验是全市人民智慧的结晶，是宝贵的财富，在今后的工作中我们一定要认真坚持并不断完善。

各位代表，过去的五年，东营经济社会全面发展，取得了辉煌的成就，实现了历史性跨越。这是在省委、省政府和市委的正确领导下，在市人大及其常委会的监督支持下，全市人民团结奋斗的结果；是人民政协、民主党派参政议政，油地军校齐心协力、携手共建的结果；是中央和省驻东营各单位积极参与、大力支持的结果。在此，我代表市人民政府向全市人民和社会各界，向所有为东营建设和发展做出贡献的同志们、朋友们，致以崇高的敬意和衷心的感谢!

同时，我们也清醒地认识到，我市经济社会发展中还有一些问题，我们的工作也存在不足。主要是：农业抵御自然灾害的能力差，工业支柱产业链条短、骨干企业少、带动能力弱，服务业比重低；自主创新能力不强，经济增长方式仍较粗放，能源资源利用效率不高；影响发展的体制和机制性障碍仍然存在；农民增收的长效机制还没有建立完善起来，城乡差距、贫富差距仍然较大，社会事业发展与广大群众的要求不相适应，生态脆弱的问题尚未得到根本改善，协调发展的任务很艰巨；一些深层次矛盾日益显现，协调社会利益关系的难度加大，影响稳定的因素仍然较多；政府职能转变还不能很好地适应市场经济发展的要求，依法行政能力需进一步提高，有些部门和工作人员作风不扎实、工作效率低，等等。对此，我们一定高度重视，采取有效措施，认真加以解决。

　　二、“十一五”时期的目标任务和工作重点

　　从今年开始，已进入第十一个五年规划时期。根据党的十六大、十六届五中全会精神和上级部署，市政府编制了《东营市国民经济和社会发展第十一个五年规划纲要(草案)》，并已经市委三届八次全委会审议。《纲要(草案)》已印发大会，请一并予以审议。

“十一五”是我市全面建设小康社会、加快现代化进程中承前启后、继往开来的重要时期，也是我们站在新起点、实现新发展的关键阶段。这一时期，经济全球化趋势将深入发展，科技进步、生产要素和产业转移加快；我国处在新一轮经济发展的上升周期，宏观发展环境日益改善；我市进入了工业化、城镇化快速推进的阶段，经济社会发展全面加速。同时，这一时期国内外竞争将更加激烈；我市虽然处在人均生产总值跨越1万美元的阶段，但由于特殊的经济社会结构，在一些方面又具有1000美元到3000美元阶段的特征，发展中仍面临着城乡差距、贫富差距较大的矛盾，资源环境对发展的约束增强的矛盾，社会文明程度、思想道德水平与经济发展不协调的矛盾，人们的维权意识增强与一些单位、企业随意侵害群众合法权益的矛盾，公务员队伍的整体素质与新形势新任务要求不相适应的矛盾，情况十分复杂。我们一定要正确把握形势，振奋精神、加倍努力，抢抓机遇、开拓进取，加快发展、科学发展，经过五年的努力，推动全市经济社会发展再上一个大的台阶。

“十一五”时期全市经济社会发展的指导思想是：以邓小平理论和“三个代表”重要思想为指导，深入贯彻党的十六大和十六届五中全会精神，以科学发展观统领经济社会发展全局，按照“一个总目标、两个率先、三个显著提高”的总体要求，坚持发展是硬道理，坚持执政为民，坚持“五位一体”的发展路子，突出以人为本，推进自主创新，完善体制机制，优化经济结构，转变增长方式，提高发展质量，实现经济社会全面协调可持续发展。

综合考虑今后五年我市经济社会发展的趋势和条件，“十一五”时期的主要预期目标是：

--综合经济实力跨上新台阶。全市生产总值年均增长15%左右，到“十一五”末突破2000亿元，五年翻一番；固定资产投资年均增长20%左右；地方财政收入年均增长15%左右；高新技术产业产值占规模以上工业总产值的比重达到25%以上，建设创新型城市。

--可持续发展能力达到新水平。建设资源节约型、环境友好型社会取得明显成效，单位生产总值能耗比“十五”末降低20%以上，全市林木覆盖率达到30%左右，形成天蓝、水清、人与自然和谐相处的优美环境。年均人口自然增长率控制在7.6‰以内。

--改革开放实现新突破。经济、社会、行政管理等各领域的改革全面推进，社会主义市场经济体制进一步完善；对外开放水平明显提高，外贸出口总值年均增长14%，实际利用外资年均增长12%。

--城乡面貌发生新变化。把中心城基本建设成功能完善的现代化区域中心城市，争创国家园林城市和中国人居环境奖；城镇化水平达到65%以上；社会主义新农村建设取得突破性进展。

--“和谐东营”建设取得新成效。社会事业全面发展，市民文明素质明显提高，政治安定，社会稳定，管理民主，争创全国文明城市；城镇登记失业率控制在2%以内，社会保障救助体系进一步完善，实现社会全面进步。

--人民生活水平实现新提高。城镇居民人均可支配收入年均增长9%左右，达到2万元以上；农民人均纯收入年均增长11%以上，力争超过8000元。

　　围绕实现上述目标，“十一五”时期重点抓好以下十个方面的工作：

(一)大力推进社会主义新农村建设。继续把解决好“三农”问题作为重中之重，按照“生产发展、生活宽裕、乡风文明、村容整洁、管理民主”的目标要求，加快建设社会主义新农村。一是着力推进农业现代化。继续培育壮大农业龙头企业，鼓励发展农村合作经济组织，提高农业产业化水平，力争到“十一五”末90%以上的农户进入产业链经营。深入实施优势农产品推广提高工程，发展高产高效粮食生产，培育壮大农业优势产业。健全完善科技、信息等农业社会化服务体系，大力推进农业科技进步，推进农业规模化、机械化，提高农业发展水平。深入实施农民培训工程，提高农民发展高效农业的能力，提高农民务工经商的能力，使每个农村劳动力掌握1门以上致富实用技术。建立健全农业标准体系、质量检测监管体系和动物疫病防控体系，加强标准化生产基地建设，提高农产品品质。加大“三荒”开发力度，积极探索拍卖“三荒”承包使用权等开发模式，力争“十一五”期间全市宜农荒碱地基本得到开发利用。树立“大国土”观念，加大海洋资源开发力度，发展海洋经济。二是着力改善农村生产生活条件。继续加强水利建设，重点搞好排河治理和田间渠改造，到“十一五”末使全市所有农田成为高标准水浇田。加快实施城乡供水一体化工程，让全市农民全部用上洁净的自来水。以沼气建设为抓手，解决农村清洁能源供应和垃圾、粪便、生活污水等处理问题。加强农村交通等设施建设和管理，不断提高乡村公路的等级和管理水平。搞好村庄规划和建设改造，全部解决无房户、危房户的住房问题。加强农村环境综合整治，推进村庄绿化美化、街道硬化，建设花园式村庄。深入推进小康文明村建设，加快发展农村教育、卫生、文化等事业，促进农村社会全面进步。三是着力发展壮大县域经济。继续实施促强扶弱等政策，鼓励支持县区、乡镇因地制宜发展特色经济，壮大优势产业，增强统筹发展能力，力争到“十一五”末有2个县区跨入全国百强行列。四是着力完善城乡统筹发展的长效机制。坚持“多予少取放活”的方针，重点在“多予”上下功夫，加大各级政府对农业和农村增加投入的力度，扩大公共财政覆盖农村的范围，强化政府对农村的公共服务，以工促农、以城带乡。引导社会资金投向“三农”，鼓励大企业投资开发农业项目。完善农村金融体系，改善农村金融环境，深入实施农村文明信用工程，鼓励各金融机构加大对“三农”的支持力度，支持农村信用社加快改革与发展。认真落实农村的各项政策，完善“三项补贴”等制度。巩固农村税费改革成果，加快推进农村综合改革，进一步减轻农民负担。完善以家庭联产承包为基础、统分结合的双层经营体制，大力转变农村经营管理方式，积极推行村企合一等促进农村经济发展的新模式。逐步统一城乡户籍制度和劳动力市场体系，采取综合措施拓宽农民就业、增收渠道。

(二)大力推进经济结构优化升级。坚持以市场为导向，膨胀经济总量，优化经济结构，转变增长方式，增强发展活力，提高经济整体素质和竞争力。

进一步壮大工业实力。坚持走新型工业化道路，稳步发展石油工业，加快山东加工制造业基地建设。按照“三个一批”的发展思路，搞好八大产业发展规划和骨干企业战略策划，注重错位发展，加大投入力度，加快企业技术改造和技术进步，改造提升石油化工、造纸、橡胶制品、纺织服装四大传统产业，加快发展壮大盐化工、精细化工、机械制造、食品加工四大优势产业，大力发展新材料、生物医药、电子信息三大高新技术产业，延长产业链条，打造产业集群；继续实施大企业带动战略，加快骨干企业膨胀发展，力争10家企业销售收入过百亿元；实施名牌战略，争创一批名牌产品。搞好东营经济开发区和临港工业园等园区规划建设，引导促进企业向园区聚集，优化产业布局。关心企业家的成长，造就一支高素质的企业家队伍。

努力提高服务业发展水平和在经济中的比重。坚持“工业强市、三产富市”，改造提升传统服务业，大力发展新兴服务业。引导商贸流通企业树立现代经营理念，提高发展水平，构筑有竞争力的现代流通产业体系。加快实施超市进乡镇、放心店进村工程。充分发挥土地多、交通便利的优势，积极争取国家在我市建设石油、粮棉油等战略物资储备基地，发展储备经济，并搞好我市重要物资储备体系建设。围绕实施黄河口生态旅游、滨海旅游、油城旅游、文化旅游四大工程，加大投入，加强旅游景点开发和配套设施建设，加大宣传促销力度，加快发展旅游业。搞好物流园区规划建设，整合物流资源，大力发展现代物流业。积极支持金融保险机构改革发展，壮大金融保险体系。大力发展信息、中介、社区服务等产业，推动房地产业健康发展。努力扩大消费特别是农村消费，增强对经济发展的拉动力。

大力发展民营经济。着力营造平等竞争的法制环境、政策环境和市场环境，引导更多的人从事民营经济。放宽市场准入，拓宽民营经济发展空间。引导民营企业不断增投入、上项目、扩规模，积极推进机制、科技、管理创新，进入主导产业链条，上档次、上水平。加强和改进对民营经济发展的服务，切实保护好民营企业的合法权益。

(三)大力推进城市和基础设施建设。围绕争创中国人居环境奖，突出“大空间、大绿地、大水面”的城市特色，着力完善城市功能，提高城市品位。根据新一轮城市总体规划，完善规划体系，构建东城、西城、垦利县城“一城三组团”的中心城发展格局。加强道路等城市基础设施建设，完善市政公用设施，搞好中心城对接区建设和城中村、居住小区的改造，加快大型科技、文化、旅游等设施的建设。搞好高层区规划建设，逐步解决“群山无峰”的问题。进一步加强城市绿化美化亮化，搞好城市公园建设，改造完善城市水系。加强城市管理，深入开展城市环境综合整治，改善市容市貌。坚持因地制宜、突出特色，加强县城和小城镇规划建设管理，加快城镇化进程。

进一步加强基础设施建设。加强海陆空立体交通体系建设，搞好东营港扩建和营运；改造提高东营永安机场，增加航线航班；推进黄大铁路建设；加快荣乌高速东营段、东港高速等公路建设改造，确立东营的交通枢纽地位。加强能源保障体系建设，完善城乡电网，鼓励建设大型电厂，积极发展热电联产。加强安全防护体系建设，规划建设黄河以北防潮堤，构建完善的防潮体系，同时搞好黄河口治理等其他防灾减灾工程建设。加强“数字化东营”建设，搞好应用开发，提高经济社会发展的信息化水平。

(四)大力推进体制改革。以转变政府职能为重点，推进行政管理体制改革。积极稳妥地推进事业单位综合配套改革。进一步深化企业改革，优化股权结构，完善法人治理结构，引导企业深化内部改革。加强企业上市工作，大力培植上市后备资源，促进具备条件的企业到境内外上市。进一步完善国有资产监管体制和监管方式，确保国有资产保值增值。继续深化投资体制改革，完善投资核准和备案制度，规范政府投资行为。深化价格改革，加强价格监管。完善市场体系，整顿和规范市场经济秩序，搞好食品、药品、农资等市场秩序专项整治。

(五)大力推进互利共赢的对外开放。坚持不懈地抓好招商引资，抓住国际国内产业转移的机遇，改进招商方式，拓宽招商渠道，提高招商引资质量和水平。强化专业招商，加强专业招商队伍建设，完善管理运行机制，提高招商成效。强化企业招商，引导鼓励企业对外合资合作。强化油地校联合招商，充分发挥区域招商引资的整体优势。强化境外招商，扩大利用外资。办好一年一度的东营经贸洽谈会，使之成为影响力很强的促进经贸科技合作的平台。根据我市资源优势和产业发展方向，加强招商项目的筛选论证，引导外来投资。加大招商项目推进落实力度，特别是加快推进大项目的实施。进一步加大投资软环境建设力度，不断提高服务水平，严肃查处侵害外来投资者合法权益的行为。大力发展对外经贸，引导企业加大国际市场开拓力度，不断扩大出口规模；根据发展需要，搞好紧缺资源和先进技术、关键设备进口；积极实施走出去战略，支持有条件的企业到境外投资办厂、承揽工程、输出劳务。坚持对外开放与对内合作相结合，充分发挥地处环渤海经济圈和山东半岛城市群的优势，在分工合作中加快发展。

(六)大力推进自主创新。提高自主创新能力，是提高科技水平和经济竞争力的关键，也是优化经济结构、转变增长方式的中心环节。要建立以企业为主体、市场为导向、产学研和油地校结合的技术创新体系，有效整合创新资源，增强自主创新能力。支持大企业建立研发中心，鼓励国内外研发机构和企业来我市研发转化科技成果。围绕石油化工、盐化工等产业发展和耐盐碱植物培育等领域，实施重点突破，研发一批对经济社会发展有较强带动作用的科技成果。建立完善科技产业孵化体系，积极发展创业风险投资，加强技术转让、技术咨询等中介服务，培育技术市场，促进科技成果向现实生产力转化。加大对科技创新的扶持力度，依法加强知识产权保护，健全创新激励机制。技术创新关键在人才，要加强人才队伍建设，大力培养、积极引进、合理使用各类人才，为产业升级提供智力支撑。

(七)大力推进区域经济社会一体化。高举团结发展的旗帜，进一步完善油地军校结合机制，推动结合向更深层次、更宽领域发展，共建美好家园。优化环境，搞好服务，支持油田、石油大学、济军生产基地的改革和发展。完善落实政策措施，搞好油田工业园和大学科技园建设，支持油田存续企业加快改制和发展壮大，支持石油大学在我市转化科技成果。深入推进区域经济社会一体化，整合油地军校的资金、技术、人才、设备、土地等资源，联手上项目、办企业，共建“和谐东营”；推进城市建设管理一体化，共同加强城市建设，完善城市功能；推进基础设施建设一体化，本着共建共用共享的原则，规划建设一批重大基础设施项目。做好油田帮扶乡村工作。油地军校结合是东营发展的独特优势和强大动力，随着结合的日益深入，我们的东营必将更加繁荣富强，人民群众的生活必将更加幸福美好!

(八)大力推进公共财政体系建设。加强财源建设，优化财政结构，不断提高地方经济的收入占整个财政收入的比重，提高县区财政收入占全市财政收入的比重。强化税收征管，确保应收尽收，把经济发展成果体现到财政收入上来。依法加强和规范政府非税收入的征管、使用，把政府非税收入全部纳入国库或财政专户，实行综合预算管理。进一步调整优化支出结构，大力压缩一般性支出，集中财力办大事，继续加大对“三农”、弱势群体、社会事业的倾斜力度。牢固树立过紧日子的思想，反对铺张浪费。深化财政改革，提高部门预算编制水平，完善国库集中收付和政府采购等制度。完善县乡财政体制，壮大县乡财政实力，逐步化解乡村债务，坚决制止发生新的债务。进一步强化审计监督，健全完善审计结论落实机制，坚决查处违规违纪行为，维护良好的财经秩序。

(九)大力推进资源节约型、环境友好型社会建设。坚持开发节约并重、节约优先，完善政策措施，依靠科技进步，大力推进节能、节水、节材、节地和发展循环经济。加快企业节能技术改造，积极发展风力发电、石油替代能源、沼气建设、地热资源开发、太阳能利用、新型墙体材料开发和生产等项目。推行城市分质供水、中水回用和一水多用，高效利用水资源。依法加强土地管理，提高土地集约化利用水平。大力发展循环经济，鼓励企业循环式生产，推动产业循环式组合，实现资源循环式利用。强化全社会的节约意识，鼓励使用节约型产品，倡导健康文明、节约资源的消费模式。深入开展创建节约型机关、节约型企业、节约型社区、节约型家庭活动，推动节约型社会建设。

进一步加强生态市建设。加大污染防治力度，严格控制新污染源的产生，加快老污染源的治理，提高污水、垃圾等处理能力，切实解决影响经济社会发展和人民健康的环境污染问题，确保人民群众喝上干净的水、呼吸新鲜的空气、吃上放心的食品。大搞植树造林，积极实施沿海防护林、中心城环城防护林和农田林网建设工程，搞好公路、水域、村镇等绿化。加大黄河三角洲自然保护区的保护力度，实施好湿地生态恢复工程。实施北水南调引黄补源工程，抑制海水入侵。深入推进生态县、环境优美乡镇、生态文明村建设，夯实生态市建设的基础。

(十)大力推进“和谐东营”建设。构建社会主义和谐社会，是经济社会发展的重要目标，也是经济社会发展的重要保障。要正确处理好加快经济发展与加快各项社会事业发展、加强物质文明建设与加强政治文明和精神文明建设的关系，正确处理好改革的力度与社会承受能力的关系，正确处理好深化企业改革、保障企业自主权、调动经营者积极性与保障职工合法权益、调动职工积极性的关系，正确处理好分配中效率与公平的关系，努力实现统筹发展、和谐发展。

着力解决人民群众最关心、最直接、最现实的利益问题。切实做好就业再就业工作，千方百计增加就业岗位，认真落实就业再就业扶持政策，完善就业服务体系，加强就业培训，努力扩大就业。合理调节收入分配，努力缓解部分社会成员收入分配差距拉大的趋势。规范企业工资支付行为，严格执行并逐步提高最低工资标准，引导企业按照工资指导线合理安排工资增长。依法查处拖欠职工工资、不为职工缴纳保险金等行为，维护职工合法权益。继续完善以“五保五救助”为主要内容的社会保障救助体系，扩大覆盖面，随着经济发展不断提高保障救助水平。扎扎实实为群众办实事办好事，围绕经济社会长远发展和改善群众生产生活条件，每年办好十件实事、实施十大工程。切实保护群众的合法权益，坚决纠正和防止征地、拆迁、改制中损害群众利益等行为。

全面发展各项社会事业。坚持教育优先发展，不断加大投入，进一步优化教育布局，提高义务教育特别是农村义务教育水平，普及高中教育，大力发展中高等职业教育和成人教育，构建以全面提高市民素质为目标、与经济社会发展相适应的教育培训体系。在抓好农民、职工、失业人员培训的同时，从今年开始，由政府提供学杂费，对未升学的初高中毕业生进行1-2年的职业教育，提高新增劳动力素质。优化医疗卫生资源配置，深化医疗卫生体制改革，构建以全面提高人民健康水平为目标的疾病预防控制和医疗服务体系。规范医疗服务收费，加强药品价格监管，强化医德医风建设，逐步推行病人住院治疗一人一房，切实解决群众看病贵及病员交叉感染等问题。加强文化设施建设，繁荣文化事业，鼓励和支持社会力量兴办民间艺术团体，发展壮大文化产业。大力开展全民健身运动，提高竞技体育水平。积极发展广播电视、新闻出版事业。坚持不懈地做好人口和计划生育工作，稳定低生育水平，提高人口素质。关心支持妇女儿童、残疾人、老年人事业，积极发展慈善事业。加强全民国防教育，抓好民兵预备役建设，巩固扩大双拥共建成果。进一步做好外事侨务、民族宗教、无线电管理、档案、气象、对台、统计、地名、史志、人防、防震和减灾救灾等工作。

加强民主法制和精神文明建设。各级政府要自觉接受人大及其常委会的监督，认真执行人大及其常委会的决议决定，支持人民政协履行政治协商、民主监督、参政议政职能，认真办理人大代表建议和政协委员提案，加强与民主党派、工商联、人民团体和社会各界的联系。扩大基层民主，保障群众依法行使民主权利。深入开展普法教育，全面推进依法治市。加强思想道德建设，广泛开展群众性精神文明创建活动，巩固和深化创建文明城市成果，深入推进“信用东营”建设，提高全社会文明程度。

进一步抓好“平安东营”建设。坚持工作重心下移，正确处理人民内部矛盾，依法加强信访工作，完善社会利益协调机制和矛盾纠纷调处机制，把各种矛盾和问题解决在基层和萌芽状态。进一步加强社会和油区治安综合治理，依法严厉打击各类犯罪行为，保持良好的社会和油区秩序。坚持不懈地抓好安全生产，认真落实安全生产责任制和责任追究制，加强安全教育，加大安全投入，狠抓事故隐患排查整改，确保全市安全生产形势持续稳定。建立健全社会预警体系和应急处理机制，完善各项应急预案，提高保障公共安全和处置突发事件的能力。

各位代表，今年是实施“十一五”规划的开局之年，做好今年的工作意义十分重大。建议今年经济社会发展的主要预期目标为：全市生产总值确保增长15%、力争增长17%；万元生产总值能耗降低4%；全社会固定资产投资增长25%；社会消费品零售总额增长15%；外贸出口总值增长15%；实际利用外资增长20%；地方财政收入增长15%；城镇居民人均可支配收入增长10%；农民人均纯收入力争增长12%；城镇登记失业率控制在2%以内；人口自然增长率控制在7.6‰以内。工作中，我们将根据“十一五”规划的总体要求，理清思路，突出重点，强化措施，狠抓落实，确保全面完成今年的目标任务，为实施“十一五”规划开好局、起好步。在全面推进各项工作的同时，继续为民办好十件实事、实施十大工程。十件实事：一是促进农民增收；二是加强村镇环境与文明建设；三是进一步搞好城乡特困救助；四是搞好安康住房建设；五是加快推进超市进乡镇、放心店进村、放心粮油食品进城区；六是实施健康关爱工程；七是建设社区健身娱乐场地；八是改造建设中心城部分农贸市场；九是建设完善全市食品药品安全监管体系；十是建设全民信息共享服务系统。十大工程：一是园区建设工程；二是东营港建设工程；三是中心城基础设施建设工程；四是城乡道路建设工程；五是旅游设施建设工程；六是公益性场馆建设工程；七是高中学校迁扩建及专业技能培训工程；八是卫生设施建设工程；九是乡镇集中供水工程；十是“平安东营”建设工程。对这十件实事、十大工程，我们要严格实行责任制，严格实行招标投标制，严格实行工程监理制，强化督查调度，确保说到做到，按期完成，取信于民!

三、加强政府自身建设

面对新形势、新任务，各级政府要加强自身建设，加快转变职能和行政管理方式，进一步提高行政效率和水平，切实担负起历史赋予的重任。

进一步加强法治政府、服务政府建设。各级政府及其部门要强化依法行政意识，树立依法行政理念，真正把依法行政贯穿于行政管理的各个环节。要严格按照法定权限和程序行使职权，全面履行好经济调节、市场监管、社会管理、公共服务职能，善于运用法律手段管理经济和社会事务。进一步加强和改善行政执法工作，转变执法方式，规范执法行为，严格执法、公正执法、文明执法。推行行政执法责任制、评议考核制和错案责任追究制，切实做到有权必有责、用权受监督、侵权须赔偿、违法要追究。深入推进政府工作提速，进一步深化行政审批制度改革，认真落实政务公开和服务承诺等制度，规范工作程序，提高行政效率。政府服务要走出机关，积极主动地为基层、企业和群众解决实际问题。

进一步加强“三个体系”建设。一是大力推进决策科学化、民主化，健全决策目标体系。改进决策规则和程序，完善重大决策调研、专家咨询论证和社会公示听证等制度，建立民意调查制度，健全群众参与、专家咨询和政府决策相结合的决策机制。对超越权限、违反法定程序造成重大损失的决策行为，严肃追究有关人员的责任。二是大力推进执行责任的具体化，健全执行责任体系。完善目标责任制和主办负责制，严格纪律，分工负责，做到工作主体清晰、责任明确、权责一致。三是大力推进考核监督的制度化、规范化，健全考核监督体系。建立科学的考核评价机制，实行工作问责制，加大监督力度。对行政不作为、影响行政效能、贻误行政工作等行为，严肃追究部门负责人和当事人的责任。

进一步加强廉政建设。坚持标本兼治、综合治理、惩防并举、注重预防的方针，建立健全教育、制度、监督并重的惩治和预防腐败体系，重点加大在土地批租、工程招投标、产权交易、政府采购等领域的反腐倡廉力度。充分发挥监察、审计等监督机关的作用，加强对行政机关运用权力、履行职责情况的监督检查。坚决纠正部门和行业不正之风，切实解决以权谋私、与民争利和损害群众利益的问题。各级政府公务员特别是领导干部都要自觉加强世界观改造，严格执行廉洁自律的各项规定，警钟长鸣，廉洁从政，执政为民。

进一步加强作风建设。深入贯彻落实公务员法，教育引导公务员加强学习，解放思想，开阔视野，不断掌握新知识，积累新经验，增长新本领，努力提高公务员队伍的整体素质。要牢记“两个务必”，进一步树立正确的政绩观，说实话、办实事、求实效，敢抓敢管，狠抓落实；坚持深入基层、深入群众调查研究，提高科学决策水平，坚决克服官僚主义和形式主义。要增强大局意识，自觉维护团结，齐心协力干事创业。要牢固树立发展依靠人民、发展为了人民、发展成果人民共享的观念，把群众冷暖安危放在心上，殚精竭虑为群众谋利益。作风建设事关政府在人民群众中的形象、事关与人民群众的血肉联系、事关行政能力建设，各级政府公务员无论职位高低、权轻权重，都必须自觉为人民掌好权、用好权，领导干部要率先垂范，切实做到对党和人民的事业高度负责，让广大群众放心满意!

各位代表!站在新起点，增创新优势，实现新发展，是时代的呼唤，是人民的重托，是历史赋予我们的神圣职责。让我们紧密团结在以胡锦涛同志为总书记的党中央周围，高举邓小平理论和“三个代表”重要思想伟大旗帜，在中共东营市委的坚强领导下，紧紧依靠全市人民，同心同德，再接再厉，求真务实，开拓进取，努力完成“十一五”规划的目标任务，共创东营更加辉煌灿烂的明天!
